# Factors influencing the initiation of adjuvant endocrine therapy in patients with estrogen receptor-positive ductal carcinoma in situ: a single institution experience

**DOI:** 10.1007/s10549-022-06735-9

**Published:** 2022-09-09

**Authors:** Julia Levy, Fady Farag, John Cole

**Affiliations:** 1 Ochsner Clinical School, University of Queensland, New Orleans, LA USA; 2 Ochsner Clinic Foundation, New Orleans, LA USA

**Keywords:** DCIS, Endocrine therapy, Adjuvant therapy, Healthcare disparities

## Abstract

**Purpose:**

This study evaluates whether race, socioeconomic status, insurance type, oncological provider type, and prior cancer treatment are associated with the suggestion and acceptance of hormonal therapy in patients with estrogen receptor (ER)-positive Ductal carcinoma in situ (DCIS). This study also assesses whether disparities exist pertaining to prescription of such medications.

**Methods:**

This single-center retrospective study included 111 patients diagnosed with DCIS between 2020 and 2021. Data collected included race, type of insurance, prescribing providers, and socioeconomic status. We used zip codes to identify the poverty levels in these areas as published in the United States Census Bureau and stratified the patients into quartiles accordingly. Chi-Square statistics were used to calculate significance levels.

**Results:**

There was no significant correlation between the intake of hormonal therapy and race (*p* = 0.60), insurance (*p* = 0.50), socioeconomic (*p* = 0.58), or providers (*p* = 0.99). 79.3% of women were offered endocrine therapy. Of those who were offered endocrine therapy, 70.8% accepted. Of patients not on hormonal therapy, 45.8% were not recommended the medications by their provider, and 54.2% declined treatment when offered.

**Conclusion:**

In this study, patients’ demographics and providers were not associated with adjuvant hormonal therapy initiation in DCIS. Our results show that abstaining from endocrine therapy in DCIS patients is both due to lack of provider recommendation and patient rejection of these medications. The wide variation in hormonal therapy treatment among ER-positive DCIS patients suggests a need for improved provider-patient communication regarding the risks and benefits of endocrine therapy in order to ensure a shared decision-making process.

## Background

In ductal carcinoma in situ (DCIS), breast epithelial cells develop into malignant but non-invasive tissue residing in the lumen of the ducts of the breast [1]. In 2022, it is estimated that 51,400 women will be diagnosed with DCIS in the USA, accounting for 17.9% of all newly diagnosed breast cancers [2]. While DCIS itself is not deadly, it is a cause of concern as it may act as a precursor to invasive breast cancers [1]. It is estimated that 20–30% of DCIS cases will progress to invasive breast cancer. However, there are currently no definitive predictive markers to identify those with DCIS who will advance into invasive breast cancer. Because of this, current guidelines suggest that DCIS patients undergo surgical resection, either alone or combined with radiation or endocrine therapy, in an effort to reduce the risk of progression to invasive disease [3].

The use of endocrine therapy in the prevention of estrogen receptor (ER)-positive DCIS progression was first reported in 1999 by the National Surgical Adjuvant Breast and Bowel Project (NSABP B-24). Tamoxifen, a selective ER modulator (SERM), was found to reduce the progression of ER-positive DCIS to invasive breast cancer by 43% [4]. The United Kingdom, Australia, New Zealand (UK-ANZ) trial found that the drug reduced rates of recurrent DCIS but not invasive breast cancer [5]. Aromatase inhibitors (AI), such as anastrozole, have also shown promise as adjuvant therapy in post-menopausal patients with DCIS. Results from the International Breast Cancer Intervention Studies II Ductal Carcinoma In Situ (IBIS-II DCIS) trial showed that anastrozole had similar efficacy to tamoxifen in reducing invasive breast cancer risk in ER-positive DCIS patients [6]. Some studies show that AIs may even be superior to tamoxifen in reducing progression to invasive breast cancer, although these medications have not shown a mortality benefit [7, 8]. Currently, the National Comprehensive Cancer Network (NCCN) guidelines recommends both tamoxifen and AIs as adjuvant therapy for women with ER-positive DCIS [3]

Despite these findings, there is still a lack of consensus regarding the benefit of endocrine therapy in ER-positive DCIS. Studies have not shown that DCIS endocrine therapy treatment improves survival, and both tamoxifen and AIs carry potential distressing side effects such as increased risk of joint pain, osteoporosis, stroke, and other thromboembolism [9]. Rates of tamoxifen initiation among DCIS patients varies widely—between 17 and 74%—across NCCN centers [10]. It has also been shown that rates of endocrine therapy initiation among ER-positive DCIS patients have been increasing, likely due to increasing acceptance of findings from these studies [11, 12]. Additionally, single-institution reports in the USA have shown that only one-half to two-thirds of DCIS patients offered tamoxifen chose to take the drug [13].

These differences in endocrine therapy initiation are largely unexplained. Several studies have investigated patient characteristics such as race, age, nuclear grade, and medical history that influence endocrine therapy initiation in DCIS. These studies found an association between endocrine therapy initiation with ER-receptor status, prior surgery, prior radiation, younger age, larger tumor size, and comedo histological growth pattern [13–19]. However, few studies have specifically addressed how both provider recommendation and patient acceptance of endocrine therapy contribute to medication initiation in women with ER-positive DCIS [13, 15]. Additionally, there are three ongoing trials currently investigating the safety and feasibility of using an active surveillance treatment strategy for low-risk DCIS in order to avoid surgery altogether. These studies suggests that the historical practice of surgical excision for all DCIS patients may not be ideal for patients deemed lower risk [20–22]. If active surveillance replaces surgical treatment for low-risk DCIS treatment, this may further complicate the use of endocrine therapy among DCIS patients. Moreover, a study by Byng et al. found that patients and providers prefer active surveillance over breast-conserving surgery and mastectomy in the treatment of low-risk DCIS [23]. Endocrine treatment preferences among patients and providers are less understood.

The primary objective of the present study is to evaluate whether personal factors, including race, socioeconomic status (SES), insurance type, oncological provider type, and prior cancer treatment are associated with the suggestion and acceptance of endocrine therapy in patients with ER-positive DCIS. Additionally, this study evaluates whether disparities exist pertaining to prescription of such medications.

## Methods

### Patient selection

This single-center retrospective study included women diagnosed with ER-positive DCIS between January 2020 and January 2021 treated at the Medical Oncology Clinic at Ochsner Medical Center in New Orleans, Louisiana. Eligibility criteria included the diagnosis of ER-positive DCIS and adequate follow-up documentation. Patients were considered ER-positive if they had ≥ 1% positive staining for ER on biopsy. Exclusion was warranted under any of the following criteria: ER-negative or unknown receptor status, current or past history of invasive breast cancer, and age < 18 years old**.** The original search identified 478 patients of which 372 were excluded for being ER-negative (n = 37) or having invasive breast cancer (*n* = 335). The final study analysis consisted of 111 patients.

### Data source

Data was collected from the institutional electronic medical record (Epic Hyperspace) and transcribed into Microsoft Excel Workbook for later analysis. The study was determined exempt by the Institutional Review Boards (IRB) of the Ochsner Clinic Foundation as a retrospective chart review that involved no diagnostic or therapeutic intervention and no direct patient contact. Informed consent from patients was not required.

Data collected included: race/ethnicity, primary insurance type, prescribing provider type, DCIS surgical resection status, zip code, and type of endocrine medication received, if any. In circumstances where patients were ER-positive and not prescribed endocrine therapy, the reason for not being prescribed therapy was also recorded through chart review. In cases where the provider did not record the reason in the patient’s chart, it was assumed that the therapy was not recommended for this patient.

We examined whether endocrine therapy initiation was associated with the following factors: primary insurance type (Medicare, Medicaid, commercial, supplemental policy, vs. other insurance), prescribing provider type (general oncologist vs. breast oncologist), poverty level, and race (non-Hispanic White, African American or Black, and Asian). Among patients who were ER-positive and not on endocrine therapy, the reason for not being on therapy was also evaluated (not recommended by provider or patient declined).

SES was assessed through assigning patient’s poverty levels using zip code data published by the 2018 United States Census Bureau. Patients were stratified into quartiles accordingly. The quartiles assigned were as follows: Below 10.6% population living in poverty (lower 25th percentile), between 10.6% and 16.4% population living poverty (25–50th), 16.4–21.9% living in poverty (50–75th), and greater than or equal to 21.9% population living in poverty (75–100th). In this case, the 75–100th percentile had the highest percentage of people living in poverty.

### Statistical Analysis

SAS analytics software version 9.4 was used to perform statistical analysis. Significance levels were calculated using a Chi-Square analysis, except for primary insurance type. Due to the low numbers of subjects in the supplemental policy insurance group (*n* = 2) and other insurance group (*n* = 1), Fisher’s exact was used to calculate significance. Due to low numbers of patients of Asian patients as well as patient with Medicare and supplemental policy, these groups were removed from Chi-square analysis in Table 2. All reported *p* values are two-sided, and *p* < 0.05 was considered statistically significant.

## Results

The study cohort consisted of 111 patients who received treatment for ER-positive DCIS at Ochsner Medical Center between January 2020 and January 2021. Patients’ age ranged from 31 to 90 years old with an average age 62.8 years old (95% CI 60.5–65.0). Patient demographic data and characteristics examined with endocrine therapy initiation is summarized in Table 1. White patients comprised 53.1% of the population, while African Americans were 45.0% of the cohort, mirroring the local population of the Greater New Orleans area. 63 (56.8%) patients received adjuvant endocrine therapy, and 48 (43.2%) did not. Among endocrine therapy users, 32 (50.8%) received anastrozole, 23 (36.5%) received tamoxifen, and 9 (12.9%) received letrozole. 19 (17.1%) of women received a mastectomy and 92 (82.9%) received a lumpectomy for treatment of DCIS. Of women who underwent mastectomy, 54 (47.4%) received adjuvant endocrine therapy compared to 38 (58.7%) of women who underwent lumpectomy. Among white patients, 31 (52.5%) took endocrine therapy, while 31 (62.0%) of African Americans received endocrine therapy. There was no statistically significant correlation between the intake of endocrine therapy and race (*p* = 0.6).

**Table 1:** Characteristics among women with ER-positive DCIS in relation to endocrine therapy use

Characteristic	Endocrine therapy		Total	*p* value
	No *n* (%)	Yes *n* (%)		
Race/ethnicity				
Non-Hispanic White	28 (47.5)	31 (52.5)	59 (53.1)	0.60
Black or African American	19 (38.0)	31 (62.0)	50 (45.0)	
Asian	1 (50.0)	1 (50.0)	2 (1.8)	
Age (years)				
< 50	6 (35.3)	11 (64.7)	17 (15.3)	0.68
50–59	7 (33.3)	14 (66.7)	21 (18.9)	
60–69	19 (51.4)	18 (48.6)	37 (33.3)	
70–79	13 (44.8)	16 (55.2)	29 (26.1)	
80 +	3 (42.9)	4 (57.1)	7 (6.3)	
Socioeconomic level (percentile)				
0-25th	13 (54.2)	11 (45.8)	24 (21.6)	0.61
25–49th	13 (44.8)	16 (55.2)	29 (25.2)	
50–74th	14 (46.7)	16 (53.3)	30 (27.0)	
75–100th	10 (35.7)	18 (64.3)	28 (25.2)	
Primary insurance type				
Commercial	19 (40.4)	28 (59.6)	47 (42.3)	0.50
Medicare	25 (45.5)	30 (54.5)	55 (49.5)	
Medicaid	2 (33.3)	4 (66.7)	6 (5.4)	
Supplemental policy	2 (100.0)	0 (0.0)	2 (1.8)	
Other	0 (0.0)	1 (100.0)	1 (0.9)	
Provider type				
General oncologist	13 (43.3)	17 (56.7)	30 (27.0)	0.99
Breast oncologist	35 (42.3)	46 (56.8)	81 (73.0)	
Total	48 (43.2)	63 (56.8)		

The majority had Medicare insurance (49.5%), or commercial insurance (42.3%). 54.5% of Medicare patients received endocrine therapy and 59.6% of commercial insurance users received adjuvant treatment. There was no significant correlation between initiation and insurance type (*p* = 0.5). Patients came from 52 different zip codes in Louisiana and Mississippi. SES-related poverty levels among these zip codes is summarized in Table 1. Poverty levels were not associated with therapy initiation (*p* = 0.58). Prescription rates between breast oncologists and general oncologists were nearly identical at 56.8% and 56.7% respectively.

89 (79.3%) of women were offered endocrine therapy by their provider. Of those who were offered endocrine therapy 63 (70.8%) accepted. Of patients not on endocrine therapy, 22 (45.8%) were not recommended the medications by their provider, and 26 (54.2%) declined treatment when offered. Reasons for abstaining from endocrine therapy are summarized in Table 2. More white women rejected endocrine therapy when offered as compared to black women. Additionally, there were more black women who were not offered endocrine therapy by their provider as compared to white women. However, neither of these results were statistically significant (*p* = 0.06). There was not a relationship between reasoning with socioeconomic status and primary insurance type.

**Table 2:** Characteristics and reasoning among women with ER-positive DCIS who were not treated with endocrine therapy

Characteristic	Reason not on endocrine therapy		Total *n*	*p* value
	Not offered by provider *n* (%)	Patient rejected *n* (%)		
Race/ethnicity				
Non-Hispanic White	10 (35.7)	18 (64.3)	28	0.06
Black or African American	12 (63.2)	7 (36.8)	19	
Socioeconomic level (percentile)				
0–25th	4 (36.4)	7 (63.6)	11	0.12
25–49th	4 (33.3)	8 (66.6)	12	
50–74th	11 (71.4)	4 (28.6)	15	
75–100th	4 (40.0)	6 (60.0)	10	
Primary insurance type				
Commercial	9 (47.4)	10 (52.6)	19	0.40
Medicare	15 (60.0)	10 (40.0)	25	
Total	22 (45.8)	26 (54.2)		

## Discussion

In this single-center study, 56.8% of women with ER-positive DCIS were initiated on endocrine therapy. While this is within limits of prior research which has shown initiation rates ranging from 17% to 74%, it is contradictory to a study by Flanagan et al. that found that facilities in the southeast region of the USA tend to prescribe endocrine therapy less (30.9%) than the national average (46.4%) [10, 15]. Factors including race, SES, insurance, and provider type were not associated with initiation.

Our results showed that 63.7% of endocrine therapy users were prescribed AIs and 36.3% were prescribed tamoxifen. This differs from prior studies showing that tamoxifen usage is more common among DCIS patients [24]. As tamoxifen and AI’s cause different side effects, medication choice is likely due to provider or patient preference. Alternatively, the increased use of AIs may be related to some newer studies showing increased efficacy of AIs as compared to tamoxifen in preventing reoccurrence in postmenopausal women with ER-positive DCIS [7, 8]. Although we did not record menopausal status, it is likely that the majority of our cohort was postmenopausal. The average age of menopause is 51 years old, which is over 10 years younger than the average participant in our study (62.8) [25]. Since AI have been shown to primarily benefit postmenopausal women, an older patient population may have contributed to the increased use of AIs in our study.

In accordance with the local demographics, our population was mostly black and non-Hispanic White, which limited our ability to apply these results to other races. Our population represented a wide variety of zip codes with poverty rates ranging from 5.9% to 32.4%. In accordance with our patient sample, the Ochsner Medical Center service area covers a large area that contains zip codes with both the highest (70053, 70113, 70114, 70117) and lowest (70448, 70447) rates of poverty in the health system [26]. Additionally, the average patient within our sample lived in a zip code with 16.50% of people living below the poverty line. Most patients receiving treatment at Ochsner Medical Center reside in Jefferson parish with 16.48% of the population living below the poverty line, thus our sample was considered representative of the local demographics [26].

Sociodemographic information such as census-tract income-based poverty level and race did not appear to be associated with endocrine therapy initiation in this study. Results from prior studies showing the differences between therapy initiation among black and white DCIS patients have been mixed. Three studies found that black women were slightly more likely to initiate endocrine therapy as compared to white women [15, 19, 24], while a study from six Kaiser Permanente (KP) hospitals reported that black women were 18% less likely to receive endocrine therapy as compared to white women [14]. Four other studies, however, did not find a relationship between initiation and race [16–18, 27]. Our study also did not find a relationship between SES and initiation which is consistent with prior studies [18, 28]. As all of the patients included in the current study had insurance, SES may have not affected the results as they would have if they were uninsured. Additionally, the lack of differences in our study may be related to our data being collected at a single-institution. This may have allowed for more routine institution guidelines that minimized differences, disparities, and access to care.

Few prior studies have reported information regarding reasons for abstaining from endocrine therapy in DCIS patients. Our data showed that among ER-positive DCIS patients not on endocrine therapy, 45.8% were not recommended the medications by their provider, and 54.2% declined treatment when offered. Reasons why medications were not offered by the provider or accepted by the patient were not recorded. Additionally, our analysis found that only 29.2% of women declined to take endocrine therapy when it was offered to them. This rejection is lower than results from a prior study which found that one-half to two-thirds of patients declined endocrine therapy when offered, [13] but higher than another study that found only 7.1% of patients rejected endocrine therapy when offered to them [15]. This discrepancy between patient acceptance among patients at different facilities is possibly due to provider behavior. Studies have shown that many physicians find explaining DCIS to patients difficult and terminology used when discussing the condition varies considerably [29, 30]. It is likely that the clinical uncertainty of the disease and controversial ideal treatment guidelines are contributing to the varying management and communication strategies. As physician recommendation and communication are strong influencing factors associated with endocrine therapy initiation, it is likely that the varied communication strategies are contributing to the discrepancy in patient acceptance [31]. Patient anxiety and confusion regarding DCIS could also be contributing factors, as these feelings are common in DCIS patients, as found by De Morgan et al. [32].

The strengths of our study included ethnic diversity and availability of ER status from patient records. Other studies that assessed endocrine therapy initiation failed to exclude ER-negative patients [14, 16, 17]. As therapy is recommended for only ER-positive patients, excluding ER-negative patients allowed us to reduce the effect from providers being unlikely to prescribe to ER-negative patients [3]. Since certain variables, such as the increased prevalence of ER negativity among black women, may affect decision making, this reduced the effects of hormone receptor status in influencing results [33]. We also specified whether patients were prescribed tamoxifen vs. AI’s which may have influenced decision making. There were limitations to our study. Our sample size was small with 111 patients which limited our ability to draw significant results. Additionally, we did not analyze patient’s family history of breast cancer, BRCA status, or genomic testing results. As these factors may increase the risk of developing invasive breast cancer, these considerations may have influenced prescriber recommendation and patient acceptance. We also were unable to adjust for patient comorbid conditions, such as a history of stroke, thromboembolism, diabetes, or osteoporosis, that may have influenced the decision to initiate endocrine therapy. Additionally, we assigned patients poverty levels based on census bureau data related to their zip code. However, these poverty levels may not have been representative of the patient’s actual SES which may have led to misclassification.

## Conclusion

Our results show that abstaining from endocrine therapy in DCIS patients is both due to lack of provider recommendation and patient rejection of these medications. Future studies may examine reasons why patients may reject endocrine therapy and reasons why providers may not offer endocrine therapy. It is important to note that a patient’s understanding of their personal risk in developing invasive breast cancer, treatment recommendations by providers, and an adequate understanding of the benefits is likely influential in a patient’s choice to initiate endocrine therapy [31, 34, 35]. The wide variation in endocrine therapy treatment among ER-positive DCIS patients suggests a need for improved provider-patient communication regarding the risks and benefits of endocrine therapy in order to ensure a shared decision-making process. Current trials are investigating active surveillance as a replacement for breast surgery in the treatment of low-risk DCIS [20–22]. If this standard of care is altered, it is possible that the use of endocrine therapy in DCIS treatment may be further complicated and create increasing uncertainty for both patients and providers alike. This emphasizes the importance of an individualized risk–benefit discussion with all patients whom may receive benefit from endocrine therapy to ensure adequate understanding. Future studies may want to investigate the benefits of patient-provider communication tools to help providers better communicate and patients better understand their personal risk of developing invasive breast cancer and the role of endocrine therapy in the treatment of DCIS so that optimal treatment is received.

## Data Availability

The datasets generated during and/or analyzed during the current study are available from the corresponding author on reasonable request.
